# Immune-Inflammatory markers and heart failure incidence and mortality: a population-based longitudinal study

**DOI:** 10.3389/fcvm.2026.1827994

**Published:** 2026-06-11

**Authors:** Siqi Li, Jie Cai, Feiyang Zhao, Liangkai Chen, Xia Han, Xue Xiao, Hui Xiong, Jinfeng Yang

**Affiliations:** 1 Cardiac Function Department, Wuhan Wuchang Hospital, Wuchang Hospital Affiliated to Wuhan University of Science and Technology, Wuhan, China; 2 School of Medicine, Health Science Center, Wuhan University of Science and Technology, Wuhan, China; 3 Department of Public Health, Wuhan Wuchang Hospital, Wuchang Hospital Affiliated to Wuhan University of Science and Technology, Wuhan, China; 4 Department of Nutrition and Food Hygiene, Hubei Key Laboratory of Food Nutrition and Safety, School of Public Health, Tongji Medical College, Huazhong University of Science and Technology, Wuhan, China

**Keywords:** heart failure, immune-inflammatory index, inflammation, mortality, UK Biobank

## Abstract

**Background:**

Inflammation is a crucial pathophysiological driver contributing to the initiation and perpetuation of heart failure (HF). This study conducted a rigorous comparison among six immune-inflammatory indices to evaluate their predictive role for HF incidence and subsequent mortality.

**Methods:**

We analyzed data from 415,263 participants free of HF and 1,952 patients with established HF from the UK Biobank. The markers assessed were the systemic immune-inflammation index (SII), systemic inflammation response index (SIRI), aggregate index of systemic inflammation (AISI), inflammatory burden index (IBI), C-reactive protein-albumin-lymphocyte (CALLY) index, and neutrophil-to-lymphocyte ratio (NLR). The outcomes were incident HF and all-cause mortality among HF patients.

**Results:**

Elevated levels of SII, SIRI, AISI, IBI, and NLR showed significant, dose-response relationships with an enhanced likelihood of HF events. The IBI demonstrated the strongest association, with participants in the highest quintile having an 86% higher risk of HF than those in the lowest quintile (HR 1.86, 95% CI: 1.75–1.97). Conversely, a higher CALLY was associated with a lower risk of HF (HR for Q5 vs. Q1: 0.56, 95% CI: 0.53–0.60). Among HF patients, SIRI, AISI, IBI, and NLR had a positive correlation with all-cause mortality, whereas the CALLY was associated with improved survival (HR 0.81, 95% CI: 0.70–0.94). ROC analyses confirmed that IBI provided the superior predictive accuracy for both incident HF and mortality.

**Conclusion:**

Immune-inflammatory markers are significant predictors of HF incidence and prognosis. The IBI emerged as the most robust biomarker, underscoring its utility for risk stratification in primary and secondary HF prevention.

## Introduction

Heart failure (HF) represents a formidable terminus of numerous cardiovascular diseases (CVD), posing an escalating global public health challenge with prevalence exceeding 64 million and climbing, fueled by demographic aging (1). Despite significant therapeutic advances, the prognosis of HF remains unsatisfactory, as evidenced by persistently high and even increasing mortality rates (2). This stark reality underscores an urgent need for improved strategies in both primary prevention, through early identification of at-risk individuals, and secondary prevention, via enhanced risk stratification among established patients.

The pathophysiological landscape of HF is increasingly recognized as profoundly shaped by chronic inflammation and immune dysregulation (3). Chronic low-grade inflammation is a key determinant of the pathological cascade leading to HF, encompassing cardiac reconstruction, progressive fibrosis, and eventual functional decompensation (4). This mechanistic insight has spurred considerable interest in the use of circulating immune-inflammatory biomarkers for risk prediction. Early investigations indicated that C-reactive protein (CRP) possesses predictive value for CVD (5). Recognized indicators which include the neutrophil-lymphocyte ratio (NLR) are powerful indicators of HF onset (6). They provide a relatively narrow view of the complex, interconnected immune-inflammatory network.

Consequently, a new generation of composite indices that integrate multiple blood cell components and acute-phase proteins has emerged to provide a more comprehensive assessment of systemic inflammatory status. These include the aggregate index of systemic inflammation (AISI), systemic immune-inflammation index (SII), systemic inflammation response index (SIRI), inflammatory burden index (IBI), and C-reactive protein-albumin-lymphocyte (CALLY) index. Individually, markers such as SII and AISI have been linked to HF incidence and adverse outcomes in various cohorts (7), whereas others, such as IBI and CALLY, show promise in predicting cardiovascular events and cancer prognosis (8, 9). However, current evidence is characterized by a critical limitation: studies typically investigate these markers in isolation and focus solely on disease onset or progression. This fragmented approach precludes a definitive, head-to-head comparison of their relative predictive power across the full spectrum of HF development, i.e., from initial occurrence in a healthy population to post-diagnosis mortality. Such a systematic evaluation is paramount for identifying the most clinically potent biomarker to guide resource allocation and intervention strategies.

Therefore, utilizing the extensive phenotypic data and long-term follow-up of the UK Biobank, this population-based longitudinal study aims to concurrently evaluate the associations of six prominent immune-inflammatory markers (SII, SIRI, AISI, IBI, CALLY index, and NLR) with the incidence of HF among initially healthy individuals and all-cause mortality among those with established HF. We hypothesize that these markers will exhibit varying strengths of association with each endpoint. By providing a comprehensive, direct comparison within a unified framework, our study seeks to identify the most robust biomarker, thereby offering valuable insights for refining risk stratification and informing future targeted anti-inflammatory strategies in the management of HF.

## Methods

### Study population

The UK Biobank recruited over 500,000 volunteers aged 40–69 from England, Scotland, and Wales. Recruitment began in 2006, lasted for more than four years, and volunteers will be followed up for the next 30 years (10). The Biobank contains extensive multidimensional data, including sociodemographic characteristics, lifestyle factors, and electronic health records. The North West Multi-center Research Ethics Committee granted ethical approval (Reference: 21/NW/0157), and each participant provided written informed consent at the time of registration.

For the purposes of this study, we initially included 502,617 participants, they were split into 500,215 participants without HF and 2,402 patients with an official diagnosis of HF based on their baseline HF medical history. We excluded participants from the HF-free group, individuals who had missing data on immune-inflammatory markers (*n* = 84,705) or lacked follow-up data for HF (*n* = 247). Similarly, among HF patients, those with missing immune-inflammatory markers (*n* = 317) or absent follow-up mortality data (*n* = 133) were excluded. The final study cohort consisted of 417,215 individuals, including 415,263 free of HF and 1,952 with established HF (Supplementary Figure S1).

### Calculation of immune-inflammatory markers

Six immune-inflammatory indices were evaluated, including NLR, SII, SIRI, AISI, IBI, and CALLY. These indices were derived from baseline blood assay data collected by the UK Biobank. Peripheral blood cell counts, CRP, and albumin levels were measured using fully automated hematology analyzers (11). Supplementary Table S1 lists the detailed calculation formulas for six immune-inflammatory indices and their corresponding field IDs.

### Outcomes

The outcomes of interest included the first occurrence of HF and all-cause mortality among HF patients. These outcomes were ascertained through the UK Biobank's linked hospital inpatients non-paper-based records and death registry data. HF events were defined based on the International Classification of Diseases (ICD-9 and ICD-10) codes and self-reported information. Detailed information regarding the outcomes definition is provided in Supplementary Table S2. All-cause mortality is the aggregate of deaths due to all particular circumstances in patients with HF, with mortality status ascertained through official death registry records. Inpatient records were accessible in England on 31 October 2022, in Scotland on 31 July 2021, and in Wales on 28 February 2018, while mortality data were available on 30 November 2022. We treated these dates as censorship when calculating follow-up duration.

### Covariates

The baseline phase's touchscreen questionnaire survey can acquire certain personal details including age, gender and ethnicity. Ethnicity was categorized as White or non-White, with the latter including individuals of mixed, Asian, Black, and other ethnic backgrounds. Socioeconomic status was assessed using the Townsend deprivation index, with higher scores indicating lower socioeconomic status (12). Educational attainment was categorized into five groups, i.e., college or university education, vocational training, high school education, lower secondary education, or other qualifications. Smoking status was classified as never, former, or current smoker. Alcohol consumption frequency was categorized as never or only on special occasions, one to three times per month, once or twice per week, three or four times per week, and daily or almost daily. Physical activity was assessed using the International Physical Activity Questionnaire (IPAQ) and categorized into low, moderate, or high levels (13).

Body mass index (BMI) can be derived from the formula of weight in kilograms divided by the square of height in meters. Estimated glomerular filtration rate (eGFR) was calculated using the 2009 Chronic Kidney Disease Epidemiology Collaboration equation, based on baseline serum creatinine concentration, age, and sex (14). Medical history of hypertension, CVD including coronary heart disease and stroke, dyslipidemia, and diabetes were determined using ICD-9, ICD-10, illness codes, and self-reported fields. Further details are provided in our prior publication (15) and in Supplementary Table S3.

### Statistical analysis

Based on the estimated numbers of the six immune-inflammatory indices, we classified the individuals into five distinct categories. We also applied Cox proportional hazards models to figure out the link between six immune-inflammatory indicators and outcomes. Additionally, the hazard ratios (HR) and corresponding 95% confidence intervals (CI) were presented to facilitate analysis. The Missing covariate data were handled using a dummy variable approach. Three models were established: Model 1 was adjusted for age, sex, ethnicity, education attainment, and the Townsend deprivation index; Model 2 further adjusted for smoking status, alcohol consumption, and IPAQ activity level; Model 3 included additional adjustments for BMI, eGFR, diabetes, hypertension, dyslipidemia, and CVD. All Cox proportional hazards models adjusted for age as a continuous variable.

The prognostic performance of the six immune-inflammatory markers for HF incidence and mortality was evaluated using receiver operating characteristic (ROC) curve analysis. The areas under the ROC curve (AUC) were compared to assess the discriminatory ability of each marker. To facilitate direct comparison of the markers, values for protective markers were inverted (multiplied by −1) to enable consistent interpretation across the models.

Additionally, stratified analyses were implemented to judge whether the associations between the immune-inflammatory markers and incident HF were consistent across subgroups defined by age, sex, smoking status, BMI, IPAQ activity level, diabetes, dyslipidemia, CVD disease, and hypertension. Finally, to verify the statistical validity of our findings, sensitivity analyses were performed that excluded participants who developed HF or died within the first year of follow-up, as well as those with cancer, together with participants having baseline evidence of acute infection (CRP >10 mg/L or white blood cell count >11 × 10⁹/L). We further assessed the incremental predictive value of each immune-inflammatory marker by calculating the integrated discrimination improvement (IDI) and net reclassification improvement (NRI) based on Model 3. All statistical analyses were conducted using R (version 4.1.3) and SAS (version 9.4). A two-tailed *P*-value <0.05 was considered statistically significant.

## Results

### Baseline characteristics

Among the 415,263 participants included in the analysis, 14,837 (3.6%) developed incident HF (Table 1). Those who developed HF were more likely to be older, male, and overweight, with a higher prevalence of long-term smoking, lower educational levels, reduced physical activity, and poorer socioeconomic status. Additionally, they were more likely to have comorbidities such as diabetes, dyslipidemia, hypertension, CVD, and renal insufficiency. Supplementary Table S4 presents the characteristics of the 1,952 patients diagnosed with HF, among whom 865 (44.3%) experienced all-cause mortality during follow-up.

**Table 1:** Baseline characteristics of the individuals without HF.

Characteristic	Total (*N* = 415,263)	HF	
		No (*N* = 400,426)	Yes (*N* = 14,837)
Age, mean (SD), years	56.53 (8.09)	56.32 (8.08)	62.10 (6.17)
Male,	191,539 (46.1)	182,197 (45.5)	9,342 (63.0)
Townsend deprivation index	−2.15 (−3.65, 0.51)	−2.17 (−3.67, 0.47)	−1.62 (−3.34, 1.62)
Ethnicity, *n* (%)			
White	391,648 (94.3)	377,617 (94.3)	14,031 (94.6)
Non-white	21,725 (5.2)	20,999 (5.2)	726 (4.9)
Unknown	1,890 (0.5)	1,810 (0.5)	80 (0.5)
Education, *n* (%)			
College or University	134,311 (32.3)	131,230 (32.8)	3,081 (20.8)
Vocational	48,851 (11.8)	46,699 (11.7)	2,152 (14.5)
Upper secondary	46,160 (11.1)	44,966 (11.2)	1,194 (8.0)
Lower secondary	110,375 (26.6)	107,003 (26.7)	3,372 (22.7)
Others	70,635 (17.0)	65,862 (16.4)	4,773 (32.2)
Unknown	4,931 (1.2)	4,666 (1.2)	265 (1.8)
Smoking status, *n* (%)			
Never	226,162 (54.5)	220,251 (55.0)	5,911 (39.8)
Former	143,337 (34.5)	136,797 (34.2)	6,540 (44.1)
Current	43,680 (10.5)	41,398 (10.3)	2,282 (15.4)
Unknown	2,084 (0.5)	1,980 (0.5)	104 (0.7)
Alcohol consumption, *n* (%)			
Daily or almost daily	84,716 (20.4)	81,518 (20.4)	3,198 (21.6)
3 or 4 times a week	95,967 (23.1)	93,140 (23.3)	2,827 (19.1)
Once or twice a week	107,123 (25.8)	103,779 (25.9)	3,344 (22.5)
1-3 times a month	45,998 (11.1)	44,473 11.1)	1,525 (10.3)
Never or special occasions	80,545 (19.4)	76,649 (19.1)	3,896 (26.3)
Unknown	914 (0.2)	867 (0.2)	47 (0.3)
IPAQ activity group			
Low	63,173 (15.2)	60,408 (15.1)	84,716 (18.6)
Moderate	136,663 (32.9)	132,143 (33.0)	95,967 (30.5)
High	135,771 (32.7)	131,556 (32.9)	107,123 (28.0)
Unknown	79,716 (19.2)	76,319 (19.0)	45,998 (22.9)
BMI, mean (SD), kg/m^2^	27.42 (4.77)	27.33 (4.7)	29.80 (5.8)
eGFR, mean (SD), ml/min/1.7m^2^	90.10 (14.15)	90.46 (13.91)	80.31 (16.66)
CVD, *n* (%)	28,144 (6.8)	24,070 (6.0)	4,074 (27.5)
Hypertension, *n* (%)	231,899 (55.8)	219,908 (54.9)	11,991 (80.8)
Diabetes, *n* (%)	25,318 (6.1)	22,550 (5.6)	2,768 (18.7)
Dyslipidemia, *n* (%)	230,874 (55.6)	219,675 (54.9)	11,199 (75.5)

### Associations between immune-inflammatory indices and incident HF

**Figure 1:**
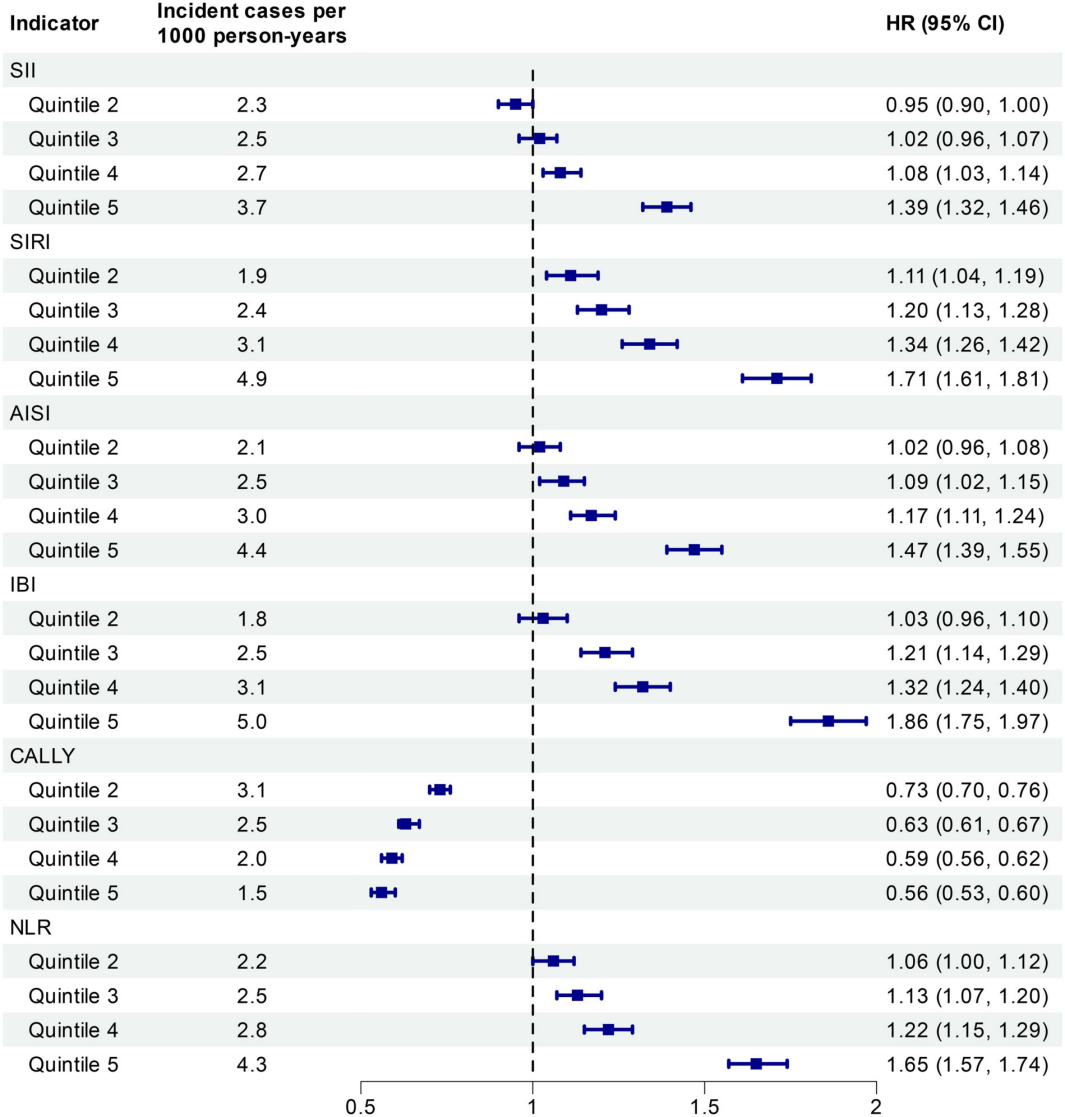
Association between six immune-inflammatory markers and the incidence of HF. Adjusted for age, sex, ethnicity, education, Townsend deprivation index, alcohol consumption, smoking status, BMI, IPAQ activity groups, eGFR, diabetes, dyslipidemia, hypertension, and CVD.

**Figure 2:**
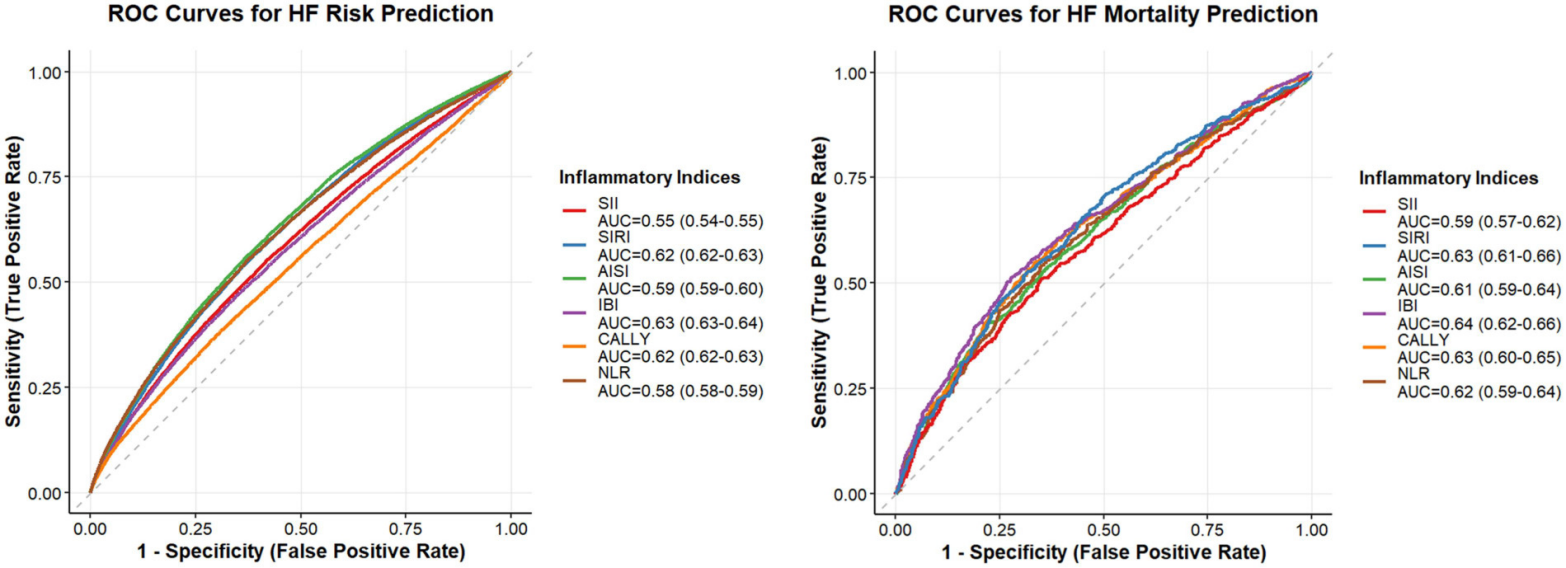
ROC curves of six immune-inflammatory biomarkers for predicting HF incidence and mortality among HF patients. For the CALLY index, values were inverted (multiplied by −1) for comparison.

The associations between the six immune-inflammatory indices and the risk of incident HF in the general population are shown in Figure 1 and Supplementary Table S5. In the fully adjusted model, elevated levels of most indices were significantly associated with an increased risk of HF in a dose-response manner. Compared with participants in the lowest quintile, those in the highest quintile of IBI had an 86% higher risk of HF (HR 1.86, 95% CI: 1.75–1.97). A similar graded positive association was observed for SII (HR 1.39, 95% CI: 1.32–1.46), SIRI (HR 1.71, 95% CI: 1.61–1.81), AISI (HR 1.47, 95% CI: 1.39–1.55), and NLR (HR 1.65, 95% CI: 1.57–1.74). For each standard deviation (SD) increment in these indices, the risk of HF increased by 2% to 8%. In contrast, the CALLY index exhibited a strong inverse association with HF risk. Participants in the highest quintile of CALLY had a 44% lower risk compared to those in the lowest quintile (HR 0.56, 95% CI: 0.53–0.60), and each SD increase in CALLY was associated with a 27% reduction in HF risk (HR 0.73, 95% CI: 0.70–0.76). The ROC curve analysis in Figure 2 showed that all six immune-inflammatory markers were predictive of HF incidence, with AUC values of 0.55 for SII, 0.62 for SIRI, 0.59 for AISI, 0.63 for IBI, 0.58 for NLR, and 0.62 for CALLY. Notably, IBI achieved the highest AUC among the inflammatory indices, at 0.63.

### Associations between immune-inflammatory indices and all-cause mortality in HF patients

**Figure 3:**
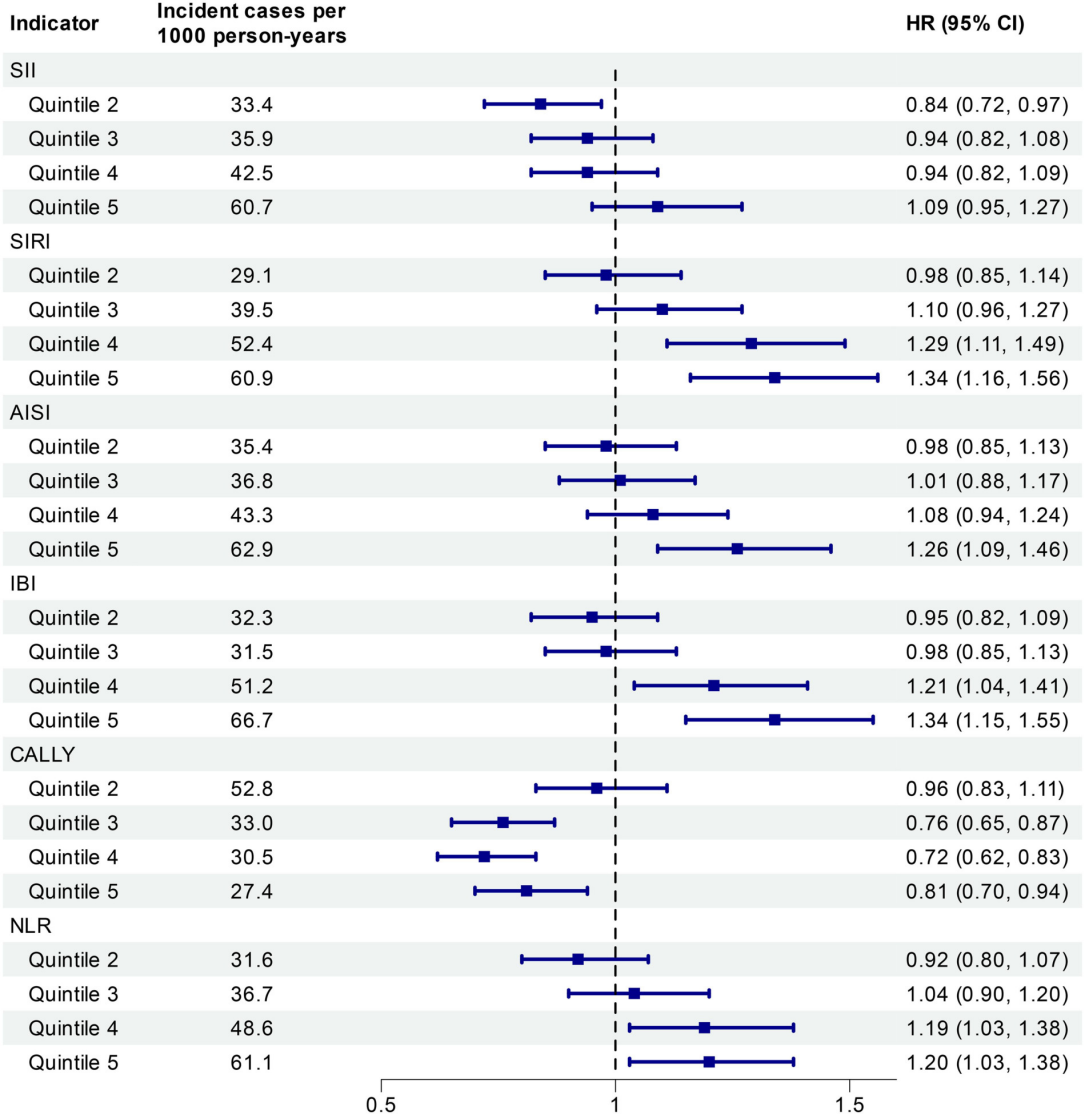
Association between six immune-inflammatory markers and the mortality of HF patients. Adjusted for age, sex, ethnicity, education, Townsend deprivation index, alcohol consumption, smoking status, BMI, IPAQ activity groups, eGFR, diabetes, dyslipidemia, hypertension, and CVD.

All indices exhibited dose-response trends with mortality risk (Figure 3 and Supplementary Table S6). Patients in the highest SIRI quintile faced 1.34 times the risk of death compared to those in the lowest quintile (HR 1.34, 95% CI: 1.16–1.56). Significant increases in mortality risk were also observed for the highest quintiles of NLR (HR 1.20, 95% CI: 1.03–1.38), AISI (HR 1.26, 95% CI: 1.09–1.46), and IBI (HR 1.34, 95% CI: 1.15–1.55). For SII, although a significant positive trend was observed (*P* for trend <0.001), the association for the highest quintile was no longer statistically significant in Model 3 (HR 1.09, 95% CI: 0.95–1.27). Each SD increase in SIRI, NLR, AISI, and IBI, mortality risk increased by 11% to 17%. Patients in the highest quintile of CALLY had a 19% lower risk of death compared to those in the lowest quintile (HR 0.81, 95% CI: 0.70–0.94), and each SD increase in CALLY was associated with a 15% risk reduction (HR 0.85, 95% CI: 0.77–0.94). The ROC curve analysis indicated that the six immune-inflammatory markers were valuable for estimating mortality risk in HF patients (Figure 2), with AUC values of 0.59 for SII, 0.63 for SIRI, 0.61 for AISI, 0.64 for IBI, 0.62 for NLR, and 0.63 for CALLY. IBI showed the optimal predictive performance, as reflected by its highest AUC of 0.64.

### Secondary analyses

In stratified analyses, BMI and comorbidities did not significantly alter the correlation between immune-inflammatory indicators and the development of HF. However, the strength of these associations was significantly modified by several factors. Specifically, compared with men, the positive associations of AISI, SIRI, and NLR with HF incidence were stronger in women (Supplementary Tables S7–S9). Age-stratified analyses showed that the associations of CALLY (Supplementary Table S10), IBI (Supplementary Table S11), and SIRI with HF incidence were significantly modified by age (*P* for interaction=0.014, 0.002, and 0.011, respectively), with stronger associations observed in participants younger than 60 years, while no significant age-related effect modification was found for AISI, NLR, or SII (Supplementary Table S12) (all *P* for interaction > 0.05). Furthermore, the associations for IBI and SII were significantly modified by smoking status and physical activity level, respectively. Sensitivity analyses, excluding individuals with baseline CRP >10 mg/L or white blood cell count >11 × 10⁹/L together with those who developed HF or died within the first year of follow-up, or those with cancer, the robust results remained (Supplementary Tables S13 and S14). The IDI and NRI were further calculated using Model 3 as the baseline model. For incident HF, addition of any inflammatory marker significantly improved the IDI. IBI showed the largest IDI and was the only marker with a significantly positive NRI, indicating superior incremental value (Supplementary Table S15). For the HF patient cohort, the sample size was limited, and the IDI/NRI estimates for mortality were unstable.

## Discussion

Drawing upon a large-sample longitudinal investigation, we systematically evaluated the associations of six immune-inflammatory markers with the incidence and mortality of HF. Our analysis included 415,263 participants free of HF and 1,952 patients diagnosed with HF at baseline. This research is groundbreaking within the current state of knowledge, and it contributes by offering a head-to-head comparison of these indices—SII, SIRI, AISI, IBI, NLR, and CALLY—in relation to HF development and outcome. We found that elevated levels of SII, SIRI, AISI, IBI, and NLR were significantly associated with an increased risk of incident HF and all-cause mortality among HF patients, demonstrating clear dose-response relationships. In contrast, the CALLY index exhibited a consistent protective effect. Notably, the IBI demonstrated superior predictive performance, achieving the highest AUCs for both incident HF and mortality, underscoring the substantial clinical translational prospect of IBI, which lies in its capacity to function as a composite biomarker for risk evaluation throughout the continuum of HF.

Our findings align with and extend previous evidence linking immune-inflammatory activation to HF pathogenesis and progression. NLR, a commonly recognized measure of systemic inflammation, has been consistently associated with cardiovascular morbidity and all-cause mortality (6), as well as the incidence of HF (16). Prior studies have reported that each unit increase in NLR corresponds to a 12% elevation in mortality risk among HF patients (17). Similarly, composite indices such as SII, SIRI, and AISI—which integrate platelet and leukocyte subsets—have been proven to predict major adverse cardiovascular events, comprising hospitalization for acute cardiovascular conditions, cardiovascular death, and stroke (18), notably among vulnerable populations like hypertensive individuals (19). When treated as continuous variables, SII and SIRI independently predict HF hospitalization and mortality, with enhanced prognostic accuracy in older adults (20). Although evidence regarding the CALLY index remains relatively scarce, previous work suggests its utility in predicting length of hospital stay in decompensated HF and cardiovascular mortality (21), with one study noting a 35% risk reduction in the highest quintile (22). Interestingly, we observed a non-significant increase in hazard for the highest CALLY quintile relative to the fourth quintile, suggesting a plateau effect. In end-stage HF, elevated albumin may reflect hemoconcentration or exogenous infusion rather than true nutritional benefit, and increased lymphocyte counts may represent reactive lymphocytosis without protective immune function (23). These factors could weaken the protective association of CALLY at its highest levels. Collectively, while NLR, SII, SIRI, and AISI have been widely examined in the context of HF, the prognostic role of CALLY in HF development is less well established, with existing literature focusing predominantly on survival outcomes.

Unlike previous reports that typically assessed one or two markers in isolation, our study directly compares six distinct indices within the same cohort, revealing IBI as the most potent predictor of HF risk and mortality. Originally developed in oncology (24), IBI has recently been implicated in cardiovascular disease; for instance, the highest IBI level was associated with a 43% increase in CVD prevalence (25), and elevated IBI predicted all-cause mortality in middle-aged and older adults (8). Our work advances this literature by demonstrating that IBI outperforms other inflammatory markers in predicting both HF incidence and prognosis, likely because it more comprehensively reflects the systemic inflammatory burden and immune dysregulation inherent in HF pathophysiology.

Stratified analyses further revealed important modifiers of these associations. The relationships of SIRI and NLR with predisposition to HF were gender-specific, being more prominent among females, suggesting sex-specific inflammatory pathways in HF development, including estrogen-mediated amplification of inflammatory responses and X-chromosome-linked immune gene dosage effects (26). In addition, smoking status modified the association for IBI, highlighting the synergistic detriment of smoking and inflammation on cardiovascular health.

The biological plausibility of our findings is supported by the distinct roles of immune cells in HF progression. Neutrophils exacerbate oxidative stress and endothelial dysfunction via peroxidase release, promoting myocardial injury and impairing cardiac function (27). Lymphocytes, through cytokine secretion, modulate inflammatory intensity and resolution, including anti-inflammatory responses that may counteract excessive inflammation (28). Platelets contribute to monocyte recruitment and vascular inflammation, accelerating myocardial fibrosis and to dysfunction (29). Hypoalbuminemia worsens fluid retention and pulmonary edema, thereby increasing HF risk (30), while acute-phase CRP release following injury can induce endothelial damage, ischemia, and coagulation activation, further compromising cardiac function (31).

The progression of HF involves both acute injury responses and chronic low-grade inflammatory (3). The IBI incorporates CRP (acute-phase reactant), neutrophils (innate immunity), and lymphocytes (adaptive immunity), thereby capturing the continuum from acute stress to chronic inflammation and immune exhaustion—a hallmark of HF-related immune imbalance (32). This multi-component design allows IBI to more sensitively reflect dynamic inflammatory changes over the course of HF, whereas single-component markers provide only partial insight. The enhanced predictive capability of IBI stems from the fact that it reflects a wider spectrum of the pathophysiological alterations within the HF-associated inflammatory network.

Several limitations should be considered. First, given that immune-inflammatory markers were measured only once at enrollment, this static assessment failed to capture their dynamic evolution over time, which may affect the observed associations and potentially reduce the reliability of risk stratification. Second, HF diagnoses were based primarily on ICD codes and self-report without imaging validation, and we could not distinguish between heart failure with reduced ejection fraction (HFrEF) and heart failure with preserved ejection fraction (HFpEF). The driving factors differ between the two: HFpEF is characterized by a more prominent systemic inflammatory state, while HFrEF is more linked to myocardial injury (33). Therefore, the predictive performance of indices such as IBI may differ between two subtypes. Third, the UK Biobank cohort is subject to healthy volunteer bias, with participants generally being healthier and of higher socioeconomic status than the general population, which may limit the generalizability of our hazard ratios to more diverse or higher-risk clinical populations. Fourth, despite adjustment for an abundance of demographic, lifestyle, and clinical variables, residual confounding from neglected components including undocumented infections and subclinical inflammation, could not be entirely ruled out. Finally, given that aging is associated with a state of low-grade chronic inflammation, which is itself an independent risk factor for HF (34), the possibility of residual confounding due to age-related chronic inflammation cannot be fully excluded despite careful adjustment for age as a continuous variable. Future studies employing age-matched designs are warranted to further validate our findings. Nevertheless, this study offers robust longitudinal evidence from a large cohort supporting the clinical relevance of easily obtainable inflammatory indices, particularly IBI and CALLY, for HF risk prediction and prognosis. The superior performance of IBI suggests its potential utility in refining risk assessment tools in both primary and secondary prevention.

## Conclusions

In this large, prospective study, we demonstrate that easily obtainable immune-inflammatory indices are potent and independent predictors of HF incidence and prognosis. Among the six markers evaluated, IBI exhibited superior predictive performance for both endpoints, suggesting it most effectively captures the systemic inflammatory dysregulation integral to HF pathogenesis. Collectively, these findings advocate for the integration of these indices, particularly the IBI, into routine risk stratification frameworks. Future research should focus on validating their utility in diverse populations and determining whether targeting these inflammatory pathways can translate into improved clinical outcomes.

## Data Availability

Publicly available datasets were analyzed in this study. This data can be found here: UK Biobank (https://www.ukbiobank.ac.uk/); Application ID: 524293.
