# Supplementary material for: Immune-Inflammatory markers and heart failure incidence and mortality: a population-based longitudinal study
Source: Front Cardiovasc Med. 2026 Jun 11;13:1827994. doi: 10.3389/fcvm.2026.1827994 (PMC13293835; doi:10.3389/fcvm.2026.1827994)
Supplement: Supplementary file 1 [file Table1.doc]

**Supplementary Online Content**

Supplementary Table 1. Definitions of immune-inflammatory markers

Supplementary Table 2. Definitions of HF

Supplementary Table 3. **Definition of covariates in UK Biobank study**

Supplementary Table 4. Baseline characteristics of the patients with HF grouped by all-cause death in the study

Supplementary Table 5. Association between six immune-inflammatory markers and the incidence of HF

Supplementary Table 6. Association between six immune-inflammatory markers and the mortality of HF patients

Supplementary Table 7. Stratified analysis of the association between AISI and HF incidence

Supplementary Table 8. Stratified analysis of the association between SIRI and HF incidence

Supplementary Table 9. Stratified analysis of the association between NLR and HF incidence

Supplementary Table 10. Stratified analysis of the association between CALLY and HF incidence

Supplementary Table 11. Stratified analysis of the association between IBI and HF incidence

Supplementary Table 12. Stratified analysis of the association between SII and HF incidence

Supplementary Table 13. Sensitivity analyses of the associations between immune-inflammatory markers and the risk of HF among the participants without HF

Supplementary Table 14. Sensitivity analyses of the associations between immune-inflammatory markers and all-cause mortality among patients with HF

Supplementary Table 15. Incremental predictive value of immune-inflammatory markers for incident HF assessed by IDI and NRI based on Model 3

Supplementary Figure 1. Flow chart of participants included in the present UK Biobank study

Supplementary Table 1. Definitions of immune-inflammatory markers

| Index Name (Abbreviation) | Calculation Formula | Field IDs |
| --- | --- | --- |
| Neutrophil-to-Lymphocyte Ratio (NLR) | NLR = NEU / LYM | NEU: 30140 (10^9^ cells/L) LYM: 30120 (10^9^ cells/L) |
| Systemic Immune-Inflammation Index (SII) | SII = (NEU × PLT) / LYM | NEU: 30140 (10^9^ cells/L)  LYM: 30120 (10^9^ cells/L)  PLT: 30080 (10^9^ cells/L) |
| Systemic Inflammation Response Index (SIRI) | SIRI = (NEU × MON) / LYM | NEU: 30140 (10^9^ cells/L)  LYM: 30120 (10^9^ cells/L)  MON: 30130 (10^9^ cells/L) |
| Aggregate Index of Systemic Inflammation (AISI) | AISI = (NEU × PLT × MON) / LYM | NEU: 30140 (10^9^ cells/L)  LYM: 30120 (10^9^ cells/L)  MON: 30130 (10^9^ cells/L)  PLT: 30080 (10^9^ cells/L) |
| Inflammatory Burden Index (IBI) | IBI = CRP × NEU / LYM | NEU: 30140 (10^9^ cells/L)  LYM: 30120 (10^9^ cells/L)  CRP: 30710 (mg/L) |
| C-reactive protein-albumin-lymphocyte (CALLY) Index | CALLY = ALB × LYM / (CRP × 10) | ALB: 30660 (g/L)  LYM: 30120 (10^9^ cells/L)  CRP: 30710 (mg/L) |

Abbreviations: NEU, neutrophils; LYM, lymphocytes; PLT, platelets; MON, monocytes; CRP, C-reactive protein; ALB, albumin

Supplementary Table 2. Definitions of HF

|  | ICD-9 | ICD-10 | Self-reported field IDs |
| --- | --- | --- | --- |
| Heart Failure | 428, 4280, 4281,4289 | I50, I500, I501, I509 | 20002 |

**Supplementary Table 3. Definition of covariates in UK Biobank study**

| Variable (UKB ID) | Collection | Description | Link |
| --- | --- | --- | --- |
| Age (21003) | Registry | Participant age at assessment centre visit, in years, obtained from NHS Primary Care Trust registries. Confirmed with participants at assessment centre. | https://biobank.ndph.ox.ac.uk/showcase/field.cgi?id=21003 |
| Sex (31) | Registry | From NHS Primary Care Trust registries. Confirmed with participants at assessment centre. | https://biobank.ndph.ox.ac.uk/showcase/field.cgi?id=31 |
| Ethnic background (21000) | Assessment centre visit: Questionnaire | Ethnic group (white, mixed, Asian/Asian British, Black/Black British, Chinese, other, PNTA) and ethnic background sub-categories. | https://biobank.ndph.ox.ac.uk/showcase/field.cgi?id=21000 |
| Townsend deprivation index (189) | Registry | Scores represent deprivation by local area, quantified by average home ownership, car ownership, household overcrowding, and employment rate, and were derived using national census data at time of recruitment. Higher scores indicate more deprived. | https://biobank.ndph.ox.ac.uk/showcase/field.cgi?id=189 |
| Smoking status (20116) | Assessment centre visit: Questionnaire | Smoking status / history: never, previous, current, PNTA | https://biobank.ndph.ox.ac.uk/showcase/field.cgi?id=20116 |
| Alcohol intake frequency (1558) | Assessment centre visit: Questionnaire | Daily, 3-4 times per week, 1-2 times per week, 1-3 times per month, special occasions only, never, PNTA | https://biobank.ndph.ox.ac.uk/showcase/field.cgi?id=1558 |
|
|
| Body mass index (21001) | Assessment centre visit: Physical measurements | Weight (kg) / height (m)^2 | https://biobank.ndph.ox.ac.uk/showcase/field.cgi?id=21001 |
| Creatinine（30700） | Biological samples visit: Blood assays | eGFR is calculated based on the obtained creatinine values, combined with variables such as age and gender. | https://biobank.ndph.ox.ac.uk/showcase/field.cgi?id=30700 |
| Physical activity (22032) | Assessment centre visit: Questionnaire | Low, Moderate, High | https://biobank.ndph.ox.ac.uk/showcase/field.cgi?id=22032 |
| Baseline of diabetes | ICD-10: E10, E11, E12, E13, E14  ICD-9: 250, 6480  Self-reported field IDs: 2443  Illness code: 1220, 1222,1223 | | |
| Baseline of cardiovascular disease | ICD-10: I20-25, I50, I500, I501, I509, I60, I61, I63, I64  ICD-9: 410, 411, 412, 413, 414, 428, 4280, 4281,4289, 430, 431, 434, 4340, 4341, 4349, 436  Self-reported field IDs: 6150, 3894, 3627, 20002, 20004, 4056 | | |
| Baseline of depression | ICD-10: F32, F33, F34, F38, F39  ICD-9: 2962, 2963, 2969, 3004, 3119  Illness code: 1286, 1531 | | |
| Baseline of hypertension | ICD-10: I10, I11, I12, I13, I15, O10, O11  ICD-9: 401, 402, 403, 404, 405  Self-reported field IDs: 6150  Illness code: 1065, 1072 | | |
| Baseline of dyslipidemia | ICD-10: E78  ICD-9: 2720, 2721, 2722, 2723, 2724, 2725, 2726, 2727, 2728, 2729  Self-reported field IDs: 1473 | | |

Supplementary Table 4. Baseline characteristics of the patients with HF grouped by all-cause death in the study

| Characteristic | Total  (N = 1,952) | All-cause death | |
| --- | --- | --- | --- |
|  |  | No (N = 1,087) | Yes (N = 865) |
| Age, mean (SD), years | 61.78 (6.28) | 60.70 (6.63) | 63.13 (5.52) |
| Male | 1,476 (75.6) | 801 (73.7) | 675 (78.0) |
| Townsend deprivation index | −1.03 (−3.17, 2.11) | −1.53 (−3.35, 1.68) | −0.66 (−2.85, 2.55) |
| Ethnicity, n (%) |  |  |  |
| White | 1,850 (94.8) | 1,032 (94.9) | 818 (94.6) |
| Non-white | 94 (4.8) | 55 (5.1) | 39 (4.5) |
| Unknown | 8 (0.4) | 0 (0.0) | 8 (0.9) |
| Education, n (%) |  |  |  |
| College or University | 366 (18.8) | 235 (21.6) | 131 (15.1) |
| Vocational | 284 (14.5) | 161 (14.8) | 123 (14.2) |
| Upper secondary | 163 (8.4) | 100 (9.2) | 63 (7.3) |
| Lower secondary | 428 (21.9) | 260 (23.9) | 168 (19.4) |
| Others | 666 (34.1) | 308 (28.3) | 358 (41.4) |
| Unknown | 45 (2.3) | 23 (2.1) | 22 (2.5) |
| Smoking status, n (%) |  |  |  |
| Never | 668 (34.2) | 449 (41.3) | 219 (25.3) |
| Former | 1,010 (51.7) | 519 (47.7) | 491 (56.8) |
| Current | 259 (13.3) | 112 (10.3) | 147 (17.0) |
| Unknown | 15 (0.8) | 7 (0.6) | 8 (0.9) |
| Alcohol consumption, n (%) |  |  |  |
| Daily or almost daily | 349 (17.9) | 198 (18.2) | 151 (17.5) |
| 3 or 4 times a week | 359 (18.4) | 227 (20.9) | 132 (15.3) |
| Once or twice a week | 430 (22.0) | 249 (22.9) | 181 (20.9) |
| 1-3 times a month | 229 (11.7) | 135 (12.4) | 94 (10.9) |
| Never or special occasions only | 580 (29.7) | 276 (25.4) | 304 (35.1) |
| Unknown | 5 (0.3) | 2 (0.2) | 3 (0.3) |
| IPAQ activity group |  |  |  |
| Low | 435 (22.3) | 201 (18.5) | 234 (27.1) |
| Moderate | 592 (30.3) | 330 (30.4) | 262 (30.3) |
| High | 476 (24.4) | 312 (28.7) | 164 (18.9) |
| Unknown | 449 (23.0) | 244 (22.4) | 205 (23.7) |
| BMI, mean (SD), kg/m^2^ | 30.14 (5.67) | 29.68 (5.27) | 30.72 (6.10) |
| eGFR, mean (SD), ml/min/1.7m^2^ | 72.99 (20.21) | 79.34 (17.21) | 65.01 (20.87) |
| Hypertension, n (%) | 1,705 (87.3) | 940 (86.5) | 765 (88.4) |
| Diabetes, n (%) | 549 (28.1) | 227 (20.9) | 322 (37.2) |
| Dyslipidemia, n (%) | 1,813 (92.9) | 1,006 (92.5) | 807 (93.3) |

Supplementary Table 5. Association between six immune-inflammatory markers and the incidence of HF

|  | Quintile 1 | Quintile 2 | Quintile 3 | Quintile 4 | Quintile 5 | *P* for trend | Per SD increment |
| --- | --- | --- | --- | --- | --- | --- | --- |
| SII |  |  |  |  |  |  |  |
| Cases/person-years | 2695/1076200 | 2526/1082550 | 2730/1082892 | 2950/1080478 | 3936/1063836 |  |  |
| Model 1 | 1.00 (ref.) | 0.95 (0.90, 1.01) | 1.03 (0.98, 1.09) | 1.12 (1.06, 1.18) | 1.46 (1.39, 1.53) | <0.001 | 1.06 (1.06, 1.07) |
| Model 2 | 1.00 (ref.) | 0.94 (0.89, 1.00) | 1.02 (0.97, 1.08) | 1.09 (1.04, 1.15) | 1.41 (1.34, 1.48) | <0.001 | 1.06 (1.06, 1.07) |
| Model 3 | 1.00 (ref.) | 0.95 (0.90, 1.00) | 1.02 (0.96, 1.07) | 1.08 (1.03, 1.14) | 1.39 (1.32, 1.46) | <0.001 | 1.05 (1.05, 1.06) |
| SIRI |  |  |  |  |  |  |  |
| Cases/person-years | 1667/1095349 | 2112/1090009 | 2602/1083778 | 3293/1073393 | 5163/1043430 |  |  |
| Model 1 | 1.00 (ref.) | 1.18 (1.11, 1.26) | 1.35 (1.27, 1.44) | 1.57 (1.48, 1.67) | 2.20 (2.08, 2.33) | <0.001 | 1.02 (1.02, 1.03) |
| Model 2 | 1.00 (ref.) | 1.16 (1.09, 1.24) | 1.31 (1.24, 1.40) | 1.52 (1.43, 1.61) | 2.07 (1.95, 2.19) | <0.001 | 1.02 (1.02, 1.03) |
| Model 3 | 1.00 (ref.) | 1.11 (1.04, 1.19) | 1.20 (1.13, 1.28) | 1.34 (1.26, 1.42) | 1.71 (1.61, 1.81) | <0.001 | 1.02 (1.02, 1.02) |
| AISI |  |  |  |  |  |  |  |
| Cases/person-years | 2030/1087327 | 2304/1086659 | 2696/1082907 | 3192/1075712 | 4615/1053354 |  |  |
| Model 1 | 1.00 (ref.) | 1.07 (1.01, 1.14) | 1.20 (1.13, 1.27) | 1.35 (1.28, 1.43) | 1.82 (1.73, 1.92) | <0.001 | 1.03 (1.02, 1.03) |
| Model 2 | 1.00 (ref.) | 1.06 (1.00, 1.12) | 1.17 (1.10, 1.24) | 1.30 (1.23, 1.38) | 1.70 (1.61, 1.80) | <0.001 | 1.03 (1.02, 1.03) |
| Model 3 | 1.00 (ref.) | 1.02 (0.96, 1.08) | 1.09 (1.02, 1.15) | 1.17 (1.11, 1.24) | 1.47 (1.39, 1.55) | <0.001 | 1.02 (1.02, 1.02) |
| IBI |  |  |  |  |  |  |  |
| Cases/person-years | 1474/1097578 | 1992/1095070 | 2745/1082917 | 3372/1071247 | 5254/1043649 |  |  |
| Model 1 | 1.00 (ref.) | 1.14 (1.06, 1.22) | 1.44 (1.35, 1.53) | 1.71 (1.61, 1.82) | 2.69 (2.54, 2.86) | <0.001 | 1.09 (1.08, 1.10) |
| Model 2 | 1.00 (ref.) | 1.11 (1.04, 1.19) | 1.39 (1.30, 1.48) | 1.60 (1.50, 1.70) | 2.42 (2.28, 2.57) | <0.001 | 1.09 (1.08, 1.09) |
| Model 3 | 1.00 (ref.) | 1.03 (0.96, 1.10) | 1.21 (1.14, 1.29) | 1.32 (1.24, 1.40) | 1.86 (1.75, 1.97) | <0.001 | 1.08 (1.08, 1.09) |
| CALLY |  |  |  |  |  |  |  |
| Cases/person-years | 5054/1046226 | 3382/1073794 | 2678/1082344 | 2131/1089591 | 1592/1094003 |  |  |
| Model 1 | 1.00 (ref.) | 0.66 (0.63, 0.69) | 0.54 (0.52, 0.57) | 0.47 (0.45, 0.49) | 0.42 (0.39, 0.44) | <0.001 | 0.58 (0.55, 0.60) |
| Model 2 | 1.00 (ref.) | 0.68 (0.65, 0.71) | 0.57 (0.54, 0.60) | 0.50 (0.48, 0.53) | 0.45 (0.43, 0.48) | <0.001 | 0.62 (0.59, 0.65) |
| Model 3 | 1.00 (ref.) | 0.73 (0.70, 0.76) | 0.63 (0.61, 0.67) | 0.59 (0.56, 0.62) | 0.56 (0.53, 0.60) | <0.001 | 0.73 (0.70, 0.76) |
| NLR |  |  |  |  |  |  |  |
| Cases/person-years | 2147/1085794 | 2411/1086731 | 2696/1082032 | 3052/1078654 | 4531/1052747 |  |  |
| Model 1 | 1.00 (ref.) | 1.09 (1.03, 1.16) | 1.18 (1.12, 1.25) | 1.28 (1.21, 1.36) | 1.77 (1.68, 1.86) | <0.001 | 1.05 (1.05, 1.06) |
| Model 2 | 1.00 (ref.) | 1.08 (1.02, 1.15) | 1.17 (1.10, 1.24) | 1.27 (1.20, 1.34) | 1.73 (1.64, 1.83) | <0.001 | 1.06 (1.05, 1.06) |
| Model 3 | 1.00 (ref.) | 1.06 (1.00, 1.12) | 1.13 (1.07, 1.20) | 1.22 (1.15, 1.29) | 1.65 (1.57, 1.74) | <0.001 | 1.05 (1.04, 1.05) |

Model 1: adjusted for age, sex, ethnicity, education level, and Townsend deprivation index;

Model 2: Model 1 + smoking status, alcohol consumption, and IPAQ activity group;

Model 3: Model 2 + for BMI, eGFR, diabetes, dyslipidemia, CVD, and hypertension.

Abbreviations: AISI, the aggregate index of systemic inflammation; BMI, body mass index; CALLY, C-reactive protein-albumin-lymphocyte; CI, confidence interval; CVD, cardiovascular disease; HF, heart failure; HR, hazard ratio; IBI, inflammatory burden index; IPAQ, International Physical Activity Questionnaire; NLR, neutrophil to lymphocyte ratio; SII, systemic immune-inflammation index; SIRI, systemic inflammation response index.

Supplementary Table 6. Association between six immune-inflammatory markers and the mortality of HF patients

|  | Quintile 1 | Quintile 2 | Quintile 3 | Quintile 4 | Quintile 5 | *P* for trend | Per SD increment |
| --- | --- | --- | --- | --- | --- | --- | --- |
| SII |  |  |  |  |  |  |  |
| Cases/person-years | 141/4560 | 150/4486 | 158/4396 | 183/4307 | 233/3838 |  |  |
| Model 1 | 1.00 (ref.) | 0.89 (0.77, 1.03) | 0.95 (0.83, 1.10) | 0.99 (0.86, 1.15) | 1.22 (1.05, 1.40) | <0.001 | 1.19 (1.14, 1.25) |
| Model 2 | 1.00 (ref.) | 0.87 (0.76, 1.01) | 0.96 (0.83, 1.10) | 0.97 (0.84, 1.12) | 1.20 (1.03, 1.38) | <0.001 | 1.18 (1.12, 1.24) |
| Model 3 | 1.00 (ref.) | 0.84 (0.72, 0.97) | 0.94 (0.82, 1.08) | 0.94 (0.82, 1.09) | 1.09 (0.95, 1.27) | <0.001 | 1.12 (1.06, 1.18) |
| SIRI |  |  |  |  |  |  |  |
| Cases/person-years | 113/4724 | 136/4669 | 169/4282 | 215/4105 | 232/3808 |  |  |
| Model 1 | 1.00 (ref.) | 0.96 (0.84, 1.11) | 1.15 (1.00, 1.33) | 1.31 (1.14, 1.51) | 1.44 (1.25, 1.67) | <0.001 | 1.23 (1.17, 1.29) |
| Model 2 | 1.00 (ref.) | 0.97 (0.84, 1.12) | 1.15 (1.00, 1.32) | 1.30 (1.13, 1.50) | 1.43 (1.24, 1.66) | <0.001 | 1.23 (1.17, 1.29) |
| Model 3 | 1.00 (ref.) | 0.98 (0.85, 1.14) | 1.10 (0.96, 1.27) | 1.29 (1.11, 1.49) | 1.34 (1.16, 1.56) | <0.001 | 1.17 (1.11, 1.23) |
| AISI |  |  |  |  |  |  |  |
| Cases/person-years | 121/4662 | 158/4467 | 161/4372 | 184/4254 | 241/3831 |  |  |
| Model 1 | 1.00 (ref.) | 0.99 (0.86, 1.14) | 1.07 (0.93, 1.23) | 1.11 (0.96, 1.27) | 1.37 (1.19, 1.58) | <0.001 | 1.15 (1.10, 1.21) |
| Model 2 | 1.00 (ref.) | 0.99 (0.86, 1.14) | 1.04 (0.91, 1.20) | 1.10 (0.95, 1.26) | 1.35 (1.17, 1.56) | <0.001 | 1.16 (1.11, 1.22) |
| Model 3 | 1.00 (ref.) | 0.98 (0.85, 1.13) | 1.01 (0.88, 1.17) | 1.08 (0.94, 1.24) | 1.26 (1.09, 1.46) | <0.001 | 1.11 (1.05, 1.16) |
| IBI |  |  |  |  |  |  |  |
| Cases/person-years | 121/4760 | 147/4555 | 142/4510 | 206/4026 | 249/3735 |  |  |
| Model 1 | 1.00 (ref.) | 1.04 (0.91, 1.20) | 1.06 (0.92, 1.23) | 1.30 (1.13, 1.51) | 1.56 (1.35, 1.80) | <0.001 | 1.17 (1.12, 1.21) |
| Model 2 | 1.00 (ref.) | 1.01 (0.88, 1.17) | 1.03 (0.89, 1.19) | 1.31 (1.13, 1.51) | 1.49 (1.28, 1.72) | <0.001 | 1.17 (1.13, 1.22) |
| Model 3 | 1.00 (ref.) | 0.95 (0.82, 1.09) | 0.98 (0.85, 1.13) | 1.21 (1.04, 1.41) | 1.34 (1.15, 1.55) | <0.001 | 1.13 (1.09, 1.18) |
| CALLY |  |  |  |  |  |  |  |
| Cases/person-years | 236/3783 | 212/4018 | 148/4482 | 140/4597 | 129/4705 |  |  |
| Model 1 | 1.00 (ref.) | 0.93 (0.80, 1.07) | 0.75 (0.65, 0.86) | 0.69 (0.60, 0.79) | 0.72 (0.62, 0.83) | <0.001 | 0.74 (0.66, 0.82) |
| Model 2 | 1.00 (ref.) | 0.93 (0.81, 1.07) | 0.73 (0.64, 0.85) | 0.69 (0.60, 0.80) | 0.73 (0.63, 0.84) | <0.001 | 0.77 (0.69, 0.85) |
| Model 3 | 1.00 (ref.) | 0.96 (0.83, 1.11) | 0.76 (0.65, 0.87) | 0.72 (0.62, 0.83) | 0.81 (0.70, 0.94) | <0.001 | 0.85 (0.77, 0.94) |
| NLR |  |  |  |  |  |  |  |
| Cases/person-years | 127/4685 | 145/4590 | 161/4392 | 202/4155 | 230/3764 |  |  |
| Model 1 | 1.00 (ref.) | 0.95 (0.83, 1.10) | 1.07 (0.93, 1.24) | 1.23 (1.06, 1.41) | 1.32 (1.15, 1.52) | <0.001 | 1.19 (1.14, 1.23) |
| Model 2 | 1.00 (ref.) | 0.97 (0.84, 1.11) | 1.08 (0.94, 1.24) | 1.23 (1.07, 1.42) | 1.32 (1.14, 1.52) | <0.001 | 1.19 (1.15, 1.24) |
| Model 3 | 1.00 (ref.) | 0.92 (0.80, 1.07) | 1.04 (0.90, 1.20) | 1.19 (1.03, 1.38) | 1.20 (1.03, 1.38) | <0.001 | 1.15 (1.10, 1.21) |

Model 1: adjusted for age, sex, ethnicity, education level, and Townsend deprivation index;

Model 2: Model 1 + smoking status, alcohol consumption, and IPAQ activity group;

Model 3: Model 2 + for BMI, eGFR, diabetes, dyslipidemia, CVD, and hypertension.

Abbreviations: AISI, the aggregate index of systemic inflammation; BMI, body mass index; CALLY, C-reactive protein-albumin-lymphocyte; CI, confidence interval; CVD, cardiovascular disease; HF, heart failure; HR, hazard ratio; IBI, inflammatory burden index; IPAQ, International Physical Activity Questionnaire; NLR, neutrophil to lymphocyte ratio; SII, systemic immune-inflammation index; SIRI, systemic inflammation response index.

Supplementary Table 7. Stratified analysis of the association between AISI and HF incidence

|  | N | Quintile 1 | Quintile 2 | Quintile 3 | Quintile 4 | Quintile 5 | *P* for interaction |
| --- | --- | --- | --- | --- | --- | --- | --- |
| Age |  |  |  |  |  |  |  |
| age<60 | 235568 | 1.00 (ref.) | 0.96 (0.86, 1.08) | 1.01 (0.90, 1.13) | 1.10 (0.99, 1.22) | 1.41 (1.28, 1.56) | 0.073 |
| age>=60 | 179695 | 1.00 (ref.) | 1.05 (0.97, 1.12) | 1.11 (1.04, 1.19) | 1.20 (1.13, 1.29) | 1.49 (1.40, 1.59) |  |
| Sex |  |  |  |  |  |  |  |
| female | 223724 | 1.00 (ref.) | 1.03 (0.90, 1.14) | 1.14 (1.04, 1.25) | 1.34 (1.20, 1.41) | 1.64 (1.50, 1.78) | <0.001 |
| male | 191539 | 1.00 (ref.) | 0.98 (0.89, 1.07) | 1.11 (1.03, 1.23) | 1.27 (1.17, 1.39) | 1.62 (1.49, 1.76) |  |
| Smoking status |  |  |  |  |  |  |  |
| never | 226162 | 1.00 (ref.) | 1.02 (0.93, 1.11) | 1.1 (1.01, 1.20) | 1.15 (1.06, 1.26) | 1.44 (1.33, 1.57) | 0.4 |
| former/current | 187017 | 1.00 (ref.) | 1.04 (0.96, 1.13) | 1.1 (1.01, 1.18) | 1.22 (1.13, 1.31) | 1.53 (1.43, 1.65) |  |
| BMI |  |  |  |  |  |  |  |
| <30 | 314740 | 1.00 (ref.) | 1.05 (0.97, 1.13) | 1.10 (1.02, 1.18) | 1.13 (1.05, 1.22) | 1.48 (1.38, 1.59) | 0.001 |
| ≥30 | 100523 | 1.00 (ref.) | 0.99 (0.89, 1.09) | 1.06 (0.97, 1.17) | 1.23 (1.12, 1.34) | 1.45 (1.33, 1.57) |  |
| IPAQ |  |  |  |  |  |  |  |
| high | 135711 | 1.00 (ref.) | 1.02 (0.91, 1.13) | 1.08 (0.97, 1.20) | 1.13 (1.02, 1.25) | 1.38 (1.25, 1.52) | 0.032 |
| low/moderate | 199836 | 1.00 (ref.) | 1.04 (0.95, 1.13) | 1.1 (1.01, 1.20) | 1.21 (1.11, 1.31) | 1.55 (1.43, 1.67) |  |
| CVD |  |  |  |  |  |  |  |
| no | 387119 | 1.00 (ref.) | 1.02 (0.95, 1.09) | 1.09 (1.02, 1.17) | 1.18 (1.11, 1.26) | 1.47 (1.38, 1.57) | 0.15 |
| yes | 28144 | 1.00 (ref.) | 1.03 (0.91, 1.16) | 1.06 (0.94, 1.19) | 1.14 (1.02, 1.27) | 1.44 (1.29, 1.6) |  |
| Hypertension |  |  |  |  |  |  |  |
| no | 183364 | 1.00 (ref.) | 0.98 (0.87, 1.11) | 1.07 (0.95, 1.21) | 1.14 (1.01, 1.29) | 1.44 (1.28, 1.61) | 0.047 |
| yes | 231899 | 1.00 (ref.) | 1.03 (0.96, 1.11) | 1.09 (1.02, 1.16) | 1.18 (1.11, 1.26) | 1.48 (1.39, 1.57) |  |
| Diabetes |  |  |  |  |  |  |  |
| no | 389945 | 1.00 (ref.) | 1.03 (0.97, 1.10) | 1.09 (1.03, 1.16) | 1.17 (1.10, 1.25) | 1.46 (1.38, 1.55) | 0.77 |
| yes | 25318 | 1.00 (ref.) | 0.96 (0.83, 1.12) | 1.04 (0.90, 1.19) | 1.17 (1.02, 1.34) | 1.46 (1.29, 1.66) |  |
| Dyslipidemia |  |  |  |  |  |  |  |
| no | 184389 | 1.00 (ref.) | 1.09 (0.97, 1.21) | 1.13 (1.01, 1.26) | 1.21 (1.08, 1.35) | 1.50 (1.35, 1.66) | 0.38 |
| yes | 230874 | 1.00 (ref.) | 0.99 (0.92, 1.07) | 1.06 (0.99, 1.14) | 1.15 (1.08, 1.23) | 1.45 (1.36, 1.54) |  |

All the Models were adjusted for age, sex, ethnicity, education level, Townsend deprivation index, smoking status, alcohol consumption, BMI, IPAQ activity group, eGFR, diabetes, dyslipidemia, CVD, and hypertension.

Supplementary Table 8. Stratified analysis of the association between SIRI and HF incidence

|  | N | Quintile 1 | Quintile 2 | Quintile 3 | Quintile 4 | Quintile 5 | *P* for interaction |
| --- | --- | --- | --- | --- | --- | --- | --- |
| Age |  |  |  |  |  |  |  |
| age<60 | 235568 | 1.00 (ref.) | 1.07 (0.95, 1.21) | 1.18 (1.05, 1.32) | 1.23 (1.1, 1.38) | 1.70 (1.53, 1.89) | 0.011 |
| age>=60 | 179695 | 1.00 (ref.) | 1.13 (1.05, 1.22) | 1.22 (1.13, 1.31) | 1.39 (1.29, 1.49) | 1.76 (1.64, 1.88) |
| Sex |  |  |  |  |  |  |  |
| female | 223724 | 1.00 (ref.) | 1.14 (1.04, 1.24) | 1.27 (1.17, 1.39) | 1.49 (1.37, 1.63) | 2.01 (1.84, 2.19) | <0.001 |
| male | 191539 | 1.00 (ref.) | 1.05 (0.96, 1.15) | 1.08 (0.99, 1.18) | 1.18 (1.08, 1.28) | 1.50 (1.38, 1.62) |
| Smoking status |  |  |  |  |  |  |  |
| never | 226162 | 1.00 (ref.) | 1.17 (1.07, 1.29) | 1.23 (1.12, 1.35) | 1.40 (1.28, 1.54) | 1.74 (1.59, 1.90) | 0.54 |
| former/current | 187017 | 1.00 (ref.) | 1.08 (0.99, 1.18) | 1.19 (1.10, 1.30) | 1.32 (1.22, 1.43) | 1.73 (1.60, 1.87) |
| BMI |  |  |  |  |  |  |  |
| <30 | 314740 | 1.00 (ref.) | 1.12 (1.04, 1.22) | 1.17 (1.09, 1.27) | 1.28 (1.19, 1.38) | 1.70 (1.58, 1.83) | 0.41 |
| ≥30 | 100523 | 1.00 (ref.) | 1.10 (0.99, 1.22) | 1.23 (1.11, 1.36) | 1.41 (1.28, 1.55) | 1.70 (1.55, 1.87) |
| IPAQ |  |  |  |  |  |  |  |
| high | 135711 | 1.00 (ref.) | 1.08 (0.96, 1.21) | 1.11 (0.99, 1.24) | 1.27 (1.13, 1.41) | 1.57 (1.42, 1.75) | 0.093 |
| low/moderate | 199836 | 1.00 (ref.) | 1.19 (1.08, 1.31) | 1.23 (1.12, 1.35) | 1.39 (1.27, 1.52) | 1.84 (1.69, 2.00) |
| CVD |  |  |  |  |  |  |  |
| no | 387119 | 1.00 (ref.) | 1.12 (1.04, 1.21) | 1.19 (1.11, 1.28) | 1.34 (1.26, 1.44) | 1.70 (1.59, 1.82) | 0.17 |
| yes | 28144 | 1.00 (ref.) | 1.07 (0.93, 1.23) | 1.20 (1.05, 1.37) | 1.29 (1.14, 1.47) | 1.68 (1.49, 1.90) |
| Hypertension |  |  |  |  |  |  |  |
| no | 183364 | 1.00 (ref.) | 1.16 (1.02, 1.32) | 1.25 (1.10, 1.42) | 1.40 (1.23, 1.59) | 1.75 (1.54, 1.98) | 0.43 |
| yes | 231899 | 1.00 (ref.) | 1.09 (1.02, 1.18) | 1.18 (1.10, 1.27) | 1.32 (1.23, 1.41) | 1.69 (1.58, 1.81) |
| Diabetes |  |  |  |  |  |  |  |
| no | 389945 | 1.00 (ref.) | 1.11 (1.03, 1.19) | 1.21 (1.13, 1.30) | 1.34 (1.26, 1.43) | 1.7 (1.59, 1.81) | 0.35 |
| yes | 25318 | 1.00 (ref.) | 1.12 (0.95, 1.32) | 1.13 (0.96, 1.32) | 1.29 (1.11, 1.50) | 1.7 (1.47, 1.96) |
| Dyslipidemia |  |  |  |  |  |  |  |
| no | 184389 | 1.00 (ref.) | 1.19 (1.06, 1.34) | 1.28 (1.14, 1.44) | 1.44 (1.29, 1.62) | 1.83 (1.64, 2.05) | 0.007 |
| yes | 230874 | 1.00 (ref.) | 1.07 (0.99, 1.16) | 1.16 (1.07, 1.24) | 1.29 (1.20, 1.38) | 1.65 (1.54, 1.76) |

All the Models were adjusted for age, sex, ethnicity, education level, Townsend deprivation index, smoking status, alcohol consumption, BMI, IPAQ activity group, eGFR, diabetes, dyslipidemia, CVD, and hypertension.

Supplementary Table 9. Stratified analysis of the association between NLR and HF incidence

|  | N | Quintile 1 | Quintile 2 | Quintile 3 | Quintile 4 | Quintile 5 | *P* for interaction |
| --- | --- | --- | --- | --- | --- | --- | --- |
| Age |  |  |  |  |  |  |  |
| age<60 | 235568 | 1.00 (ref.) | 1.01 (0.90, 1.12) | 1.01 (0.91, 1.13) | 1.20 (1.08, 1.34) | 1.63 (1.47, 1.80) | 0.073 |
| age>=60 | 179695 | 1.00 (ref.) | 1.08 (1.01, 1.16) | 1.18 (1.11, 1.27) | 1.23 (1.16, 1.32) | 1.69 (1.59, 1.80) |  |
| Sex |  |  |  |  |  |  |  |
| female | 223724 | 1.00 (ref.) | 1.09 (0.99, 1.19) | 1.21 (1.11, 1.32) | 1.32 (1.21, 1.44) | 1.92 (1.77, 2.09) | <0.001 |
| male | 191539 | 1.00 (ref.) | 1.03 (0.95, 1.11) | 1.07 (0.99, 1.15) | 1.14 (1.06, 1.23) | 1.51 (1.41, 1.62) |  |
| Smoking status |  |  |  |  |  |  |  |
| never | 226162 | 1.00 (ref.) | 1.08 (0.98, 1.18) | 1.13 (1.03, 1.23) | 1.21 (1.11, 1.32) | 1.67 (1.54, 1.81) | 0.62 |
| former/current | 187017 | 1.00 (ref.) | 1.06 (0.98, 1.14) | 1.14 (1.06, 1.23) | 1.23 (1.14, 1.32) | 1.65 (1.54, 1.77) |  |
| BMI |  |  |  |  |  |  |  |
| <30 | 314740 | 1.00 (ref.) | 1.03 (0.95, 1.11) | 1.07 (0.99, 1.15) | 1.17 (1.09, 1.26) | 1.56 (1.46, 1.67) | 0.14 |
| >=30 | 100523 | 1.00 (ref.) | 1.12 (1.02, 1.23) | 1.23 (1.12, 1.34) | 1.29 (1.18, 1.41) | 1.77 (1.63, 1.93) |  |
| IPAQ |  |  |  |  |  |  |  |
| high | 135711 | 1.00 (ref.) | 1.15 (1.03, 1.28) | 1.20 (1.08, 1.33) | 1.21 (1.09, 1.34) | 1.61 (1.46, 1.77) | 0.04 |
| low/moderate | 199836 | 1.00 (ref.) | 1.06 (0.98, 1.16) | 1.14 (1.05, 1.24) | 1.23 (1.13, 1.33) | 1.72 (1.60, 1.86) |  |
| CVD |  |  |  |  |  |  |  |
| no | 387119 | 1.00 (ref.) | 1.03 (0.97, 1.10) | 1.10 (1.03, 1.18) | 1.21 (1.13, 1.29) | 1.61 (1.51, 1.71) | 0.86 |
| yes | 28144 | 1.00 (ref.) | 1.16 (1.03, 1.30) | 1.23 (1.10, 1.38) | 1.26 (1.12, 1.41) | 1.78 (1.61, 1.98) |  |
| Hypertension |  |  |  |  |  |  |  |
| no | 183364 | 1.00 (ref.) | 1.11 (0.98, 1.25) | 1.03 (0.91, 1.17) | 1.25 (1.11, 1.41) | 1.63 (1.45, 1.83) | 0.49 |
| yes | 231899 | 1.00 (ref.) | 1.05 (0.98, 1.12) | 1.16 (1.09, 1.24) | 1.21 (1.14, 1.29) | 1.66 (1.57, 1.76) |  |
| Diabetes |  |  |  |  |  |  |  |
| no | 389945 | 1.00 (ref.) | 1.07 (1.00, 1.14) | 1.11 (1.05, 1.19) | 1.21 (1.14, 1.28) | 1.63 (1.54, 1.73) | 0.63 |
| yes | 25318 | 1.00 (ref.) | 0.99 (0.86, 1.14) | 1.21 (1.06, 1.39) | 1.26 (1.11, 1.44) | 1.71 (1.51, 1.93) |  |
| Dyslipidemia |  |  |  |  |  |  |  |
| no | 184389 | 1.00 (ref.) | 1.06 (0.94, 1.19) | 1.16 (1.04, 1.30) | 1.26 (1.13, 1.41) | 1.64 (1.48, 1.82) | 0.27 |
| yes | 230874 | 1.00 (ref.) | 1.06 (0.99, 1.13) | 1.12 (1.05, 1.20) | 1.20 (1.13, 1.28) | 1.65 (1.55, 1.75) |  |

All the Models were adjusted for age, sex, ethnicity, education level, Townsend deprivation index, smoking status, alcohol consumption, BMI, IPAQ activity group, eGFR, diabetes, dyslipidemia, CVD, and hypertension.

Supplementary Table 10. Stratified analysis of the association between CALLY and HF incidence

|  | N | Quintile 1 | Quintile 2 | Quintile 3 | Quintile 4 | Quintile 5 | *P* for interaction |
| --- | --- | --- | --- | --- | --- | --- | --- |
| Age |  |  |  |  |  |  |  |
| age<60 | 235568 | 1.00 (ref.) | 0.70 (0.64, 0.76) | 0.61 (0.55, 0.67) | 0.58 (0.52, 0.64) | 0.54 (0.48, 0.60) | 0.014 |
| age>=60 | 179695 | 1.00 (ref.) | 0.74 (0.70, 0.78) | 0.65 (0.61, 0.68) | 0.60 (0.56, 0.64) | 0.57 (0.53, 0.61) |  |
| Sex |  |  |  |  |  |  |  |
| female | 223724 | 1.00 (ref.) | 0.72 (0.67, 0.77) | 0.62 (0.57, 0.67) | 0.59 (0.54, 0.65) | 0.54 (0.49, 0.60) | 0.088 |
| male | 191539 | 1.00 (ref.) | 0.73 (0.69, 0.77) | 0.64 (0.60, 0.68) | 0.59 (0.55, 0.63) | 0.57 (0.53, 0.62) |  |
| Smoking status |  |  |  |  |  |  |  |
| never | 226162 | 1.00 (ref.) | 0.72 (0.67, 0.77) | 0.63 (0.58, 0.68) | 0.57 (0.53, 0.62) | 0.59 (0.54, 0.64) | 0.45 |
| former/current | 187017 | 1.00 (ref.) | 0.73 (0.69, 0.77) | 0.63 (0.59, 0.67) | 0.59 (0.56, 0.64) | 0.53 (0.49, 0.58) |  |
| BMI |  |  |  |  |  |  |  |
| <30 | 314740 | 1.00 (ref.) | 0.73 (0.68, 0.77) | 0.64 (0.60, 0.68) | 0.57 (0.53, 0.60) | 0.55 (0.51, 0.59) | 0.3 |
| >=30 | 100523 | 1.00 (ref.) | 0.73 (0.69, 0.78) | 0.62 (0.58, 0.67) | 0.66 (0.60, 0.72) | 0.54 (0.48, 0.62) |  |
| IPAQ |  |  |  |  |  |  |  |
| high | 135711 | 1.00 (ref.) | 0.78 (0.72, 0.85) | 0.67 (0.61, 0.73) | 0.61 (0.55, 0.67) | 0.58 (0.52, 0.65) | 0.38 |
| low/moderate | 199836 | 1.00 (ref.) | 0.71 (0.66, 0.75) | 0.63 (0.59, 0.67) | 0.57 (0.53, 0.62) | 0.56 (0.52, 0.61) |  |
| CVD |  |  |  |  |  |  |  |
| no | 387119 | 1.00 (ref.) | 0.72 (0.69, 0.76) | 0.63 (0.60, 0.67) | 0.59 (0.55, 0.62) | 0.56 (0.53, 0.60) | 0.07 |
| yes | 28144 | 1.00 (ref.) | 0.75 (0.69, 0.81) | 0.65 (0.59, 0.71) | 0.61 (0.56, 0.68) | 0.57 (0.51, 0.64) |  |
| Hypertension |  |  |  |  |  |  |  |
| no | 183364 | 1.00 (ref.) | 0.74 (0.67, 0.82) | 0.63 (0.56, 0.70) | 0.59 (0.53, 0.67) | 0.58 (0.51, 0.66) | 0.45 |
| yes | 231899 | 1.00 (ref.) | 0.73 (0.69, 0.76) | 0.64 (0.61, 0.67) | 0.59 (0.56, 0.63) | 0.56 (0.52, 0.60) |  |
| Diabetes |  |  |  |  |  |  |  |
| no | 389945 | 1.00 (ref.) | 0.72 (0.69, 0.76) | 0.63 (0.60, 0.66) | 0.57 (0.54, 0.61) | 0.56 (0.53, 0.60) | 0.83 |
| yes | 25318 | 1.00 (ref.) | 0.77 (0.69, 0.85) | 0.68 (0.61, 0.76) | 0.70 (0.62, 0.79) | 0.57 (0.50, 0.66) |  |
| Dyslipidemia |  |  |  |  |  |  |  |
| no | 184389 | 1.00 (ref.) | 0.75 (0.68, 0.82) | 0.65 (0.59, 0.72) | 0.57 (0.52, 0.63) | 0.55 (0.49, 0.61) | 0.55 |
| yes | 230874 | 1.00 (ref.) | 0.72 (0.69, 0.76) | 0.63 (0.60, 0.67) | 0.60 (0.57, 0.64) | 0.57 (0.54, 0.62) |  |

All the Models were adjusted for age, sex, ethnicity, education level, Townsend deprivation index, smoking status, alcohol consumption, BMI, IPAQ activity group, eGFR, diabetes, dyslipidemia, CVD, and hypertension.

Supplementary Table 11. Stratified analysis of the association between IBI and HF incidence

|  | N | Quintile 1 | Quintile 2 | Quintile 3 | Quintile 4 | Quintile 5 | *P* for interaction |
| --- | --- | --- | --- | --- | --- | --- | --- |
| Age |  |  |  |  |  |  |  |
| age<60 | 235568 | 1.00 (ref.) | 1.08 (0.95, 1.23) | 1.28 (1.13, 1.44) | 1.32 (1.17, 1.49) | 1.99 (1.77, 2.23) | 0.002 |
| age>=60 | 179695 | 1.00 (ref.) | 1.01 (0.94, 1.1) | 1.20 (1.11, 1.29) | 1.32 (1.23, 1.42) | 1.81 (1.69, 1.95) |  |
| Sex |  |  |  |  |  |  |  |
| female | 223724 | 1.00 (ref.) | 1.08 (0.96, 1.22) | 1.29 (1.15, 1.44) | 1.41 (1.27, 1.57) | 2.02 (1.82, 2.24) | 0.002 |
| male | 191539 | 1.00 (ref.) | 1.00 (0.92, 1.09) | 1.17 (1.09, 1.27) | 1.28 (1.18, 1.38) | 1.78 (1.65, 1.92) |  |
| Smoking status |  |  |  |  |  |  |  |
| never | 226162 | 1.00 (ref.) | 0.98 (0.89, 1.08) | 1.17 (1.06, 1.28) | 1.24 (1.13, 1.36) | 1.75 (1.60, 1.92) | 0.034 |
| former/current | 187017 | 1.00 (ref.) | 1.07 (0.98, 1.18) | 1.27 (1.16, 1.39) | 1.42 (1.31, 1.55) | 2.03 (1.87, 2.21) |  |
| BMI |  |  |  |  |  |  |  |
| <30 | 314740 | 1.00 (ref.) | 1.03 (0.95, 1.11) | 1.25 (1.16, 1.34) | 1.31 (1.22, 1.41) | 1.90 (1.77, 2.04) | 0.58 |
| >=30 | 100523 | 1.00 (ref.) | 1.11 (0.95, 1.29) | 1.23 (1.07, 1.41) | 1.40 (1.22, 1.60) | 1.90 (1.66, 2.16) |  |
| IPAQ |  |  |  |  |  |  |  |
| high | 135711 | 1.00 (ref.) | 1.06 (0.94, 1.19) | 1.30 (1.16, 1.45) | 1.40 (1.25, 1.57) | 1.81 (1.62, 2.02) | 0.162 |
| low/moderate | 199836 | 1.00 (ref.) | 0.99 (0.89, 1.09) | 1.16 (1.06, 1.27) | 1.28 (1.17, 1.4) | 1.86 (1.71, 2.03) |  |
| CVD |  |  |  |  |  |  |  |
| no | 387119 | 1.00 (ref.) | 1.03 (0.95, 1.12) | 1.22 (1.13, 1.31) | 1.32 (1.22, 1.42) | 1.87 (1.74, 2.00) | 0.043 |
| yes | 28144 | 1.00 (ref.) | 1.03 (0.9, 1.17) | 1.19 (1.05, 1.34) | 1.32 (1.17, 1.48) | 1.80 (1.61, 2.02) |  |
| Hypertension |  |  |  |  |  |  |  |
| no | 183364 | 1.00 (ref.) | 1.08 (0.95, 1.23) | 1.14 (1.00, 1.29) | 1.27 (1.12, 1.44) | 1.90 (1.68, 2.15) | 0.76 |
| yes | 231899 | 1.00 (ref.) | 1.02 (0.94, 1.10) | 1.23 (1.14, 1.33) | 1.33 (1.24, 1.43) | 1.85 (1.72, 1.98) |  |
| Diabetes |  |  |  |  |  |  |  |
| no | 389945 | 1.00 (ref.) | 1.01 (0.94, 1.09) | 1.22 (1.14, 1.31) | 1.31 (1.22, 1.4) | 1.87 (1.75, 2.00) | 0.68 |
| yes | 25318 | 1.00 (ref.) | 1.13 (0.95, 1.34) | 1.17 (0.99, 1.38) | 1.36 (1.16, 1.59) | 1.79 (1.54, 2.09) |  |
| Dyslipidemia |  |  |  |  |  |  |  |
| no | 184389 | 1.00 (ref.) | 1.05 (0.94, 1.19) | 1.33 (1.19, 1.50) | 1.35 (1.20, 1.52) | 1.96 (1.75, 2.19) | 0.53 |
| yes | 230874 | 1.00 (ref.) | 1.01 (0.93, 1.10) | 1.16 (1.07, 1.25) | 1.29 (1.19, 1.39) | 1.79 (1.67, 1.93) |  |

All the Models were adjusted for age, sex, ethnicity, education level, Townsend deprivation index, smoking status, alcohol consumption, BMI, IPAQ activity group, eGFR, diabetes, dyslipidemia, CVD, and hypertension.

Supplementary Table 12. Stratified analysis of the association between SII and HF incidence

|  | N | Quintile 1 | Quintile 2 | Quintile 3 | Quintile 4 | Quintile 5 | *P* for interaction |
| --- | --- | --- | --- | --- | --- | --- | --- |
| Age |  |  |  |  |  |  |  |
| age<60 | 235568 | 1.00 (ref.) | 0.88 (0.79, 0.98) | 0.96 (0.86, 1.06) | 1.01 (0.91, 1.12) | 1.33 (1.21, 1.47) | 0.65 |
| age>=60 | 179695 | 1.00 (ref.) | 0.97 (0.91, 1.03) | 1.03 (0.97, 1.09) | 1.10 (1.03, 1.17) | 1.40 (1.32, 1.48) |
| Sex |  |  |  |  |  |  |  |
| female | 223724 | 1.00 (ref.) | 0.97 (0.88, 1.06) | 1.05 (0.96, 1.15) | 1.18 (1.08, 1.29) | 1.49 (1.37, 1.61) | 0.005 |
| male | 191539 | 1.00 (ref.) | 0.94 (0.88, 1.00) | 1.00 (0.94, 1.07) | 1.03 (0.96, 1.10) | 1.34 (1.26, 1.43) |
| Smoking status |  |  |  |  |  |  |  |
| never | 226162 | 1.00 (ref.) | 0.93 (0.86, 1.01) | 0.99 (0.91, 1.08) | 1.06 (0.98, 1.15) | 1.33 (1.23, 1.44) | 0.086 |
| former/current | 187017 | 1.00 (ref.) | 0.96 (0.89, 1.03) | 1.04 (0.97, 1.11) | 1.11 (1.03, 1.18) | 1.44 (1.35, 1.54) |
| BMI |  |  |  |  |  |  |  |
| <30 | 314740 | 1.00 (ref.) | 0.92 (0.86, 0.99) | 1.00 (0.93, 1.07) | 1.03 (0.96, 1.10) | 1.34 (1.25, 1.43) | 0.055 |
| ≥30 | 100523 | 1.00 (ref.) | 0.98 (0.90, 1.07) | 1.06 (0.97, 1.15) | 1.17 (1.07, 1.27) | 1.45 (1.34, 1.57) |
| IPAQ |  |  |  |  |  |  |  |
| high | 135711 | 1.00 (ref.) | 1.04 (0.94, 1.14) | 1.07 (0.97, 1.18) | 1.09 (0.99, 1.20) | 1.28 (1.17, 1.41) | 0.002 |
| low/moderate | 199836 | 1.00 (ref.) | 0.96 (0.89, 1.04) | 1.02 (0.94, 1.10) | 1.09 (1.01, 1.17) | 1.47 (1.37, 1.58) |
| CVD |  |  |  |  |  |  |  |
| no | 387119 | 1.00 (ref.) | 0.94 (0.88, 1.00) | 1.00 (0.94, 1.06) | 1.08 (1.02, 1.15) | 1.38 (1.30, 1.46) | 0.85 |
| yes | 28144 | 1.00 (ref.) | 0.96 (0.86, 1.07) | 1.08 (0.97, 1.19) | 1.08 (0.97, 1.19) | 1.40 (1.27, 1.54) |
| Hypertension |  |  |  |  |  |  |  |
| no | 183364 | 1.00 (ref.) | 0.87 (0.77, 0.98) | 0.95 (0.85, 1.07) | 0.98 (0.88, 1.11) | 1.31 (1.17, 1.46) | 0.43 |
| yes | 231899 | 1.00 (ref.) | 0.97 (0.91, 1.03) | 1.04 (0.98, 1.10) | 1.11 (1.04, 1.17) | 1.41 (1.33, 1.49) |
| Diabetes |  |  |  |  |  |  |  |
| no | 389945 | 1.00 (ref.) | 0.94 (0.88, 1.00) | 1.00 (0.94, 1.06) | 1.06 (1.00, 1.12) | 1.36 (1.28, 1.43) | 0.085 |
| yes | 25318 | 1.00 (ref.) | 0.98 (0.86, 1.11) | 1.10 (0.97, 1.25) | 1.18 (1.05, 1.34) | 1.49 (1.33, 1.68) |
| Dyslipidemia |  |  |  |  |  |  |  |
| no | 184389 | 1.00 (ref.) | 0.94 (0.84, 1.05) | 1.00 (0.90, 1.12) | 1.10 (0.99, 1.23) | 1.30 (1.17, 1.44) | 0.41 |
| yes | 230874 | 1.00 (ref.) | 0.95 (0.89, 1.01) | 1.02 (0.96, 1.09) | 1.07 (1.01, 1.14) | 1.41 (1.33, 1.49) |

All the Models were adjusted for age, sex, ethnicity, education level, Townsend deprivation index, smoking status, alcohol consumption, BMI, IPAQ activity group, eGFR, diabetes, dyslipidemia, CVD, and hypertension.

Supplementary Table 13. Sensitivity analyses of the associations between immune-inflammatory markers and the risk of HF among the participants without HF

| Analysis  (N = 414184) | HR (95% CI) | | | | |
| --- | --- | --- | --- | --- | --- |
|  | Quintile 1 | Quintile 2 | Quintile 3 | Quintile 4 | Quintile 5 |
| SII | 1.00 (ref.) | 0.95 (0.90, 1.00) | 1.02 (0.97, 1.08) | 1.06 (1.00, 1.12) | 1.29 (1.22, 1.36) |
| SIRI | 1.00 (ref.) | 1.13 (1.05, 1.20) | 1.21 (1.14, 1.29) | 1.37 (1.28, 1.46) | 1.67 (1.57, 1.78) |
| AISI | 1.00 (ref.) | 1.04 (0.98, 1.11) | 1.10 (1.04, 1.17) | 1.19 (1.12, 1.26) | 1.41 (1.33, 1.49) |
| IBI | 1.00 (ref.) | 1.01 (0.94, 1.08) | 1.17 (1.10, 1.25) | 1.26 (1.18, 1.35) | 1.64 (1.54, 1.75) |
| CALLY | 1.00 (ref.) | 0.78 (0.74, 0.82) | 0.69 (0.66, 0.73) | 0.65 (0.62, 0.69) | 0.64 (0.60, 0.68) |
| NLR | 1.00 (ref.) | 1.07 (1.01, 1.14) | 1.13 (1.07, 1.20) | 1.22 (1.15, 1.29) | 1.58 (1.49, 1.67) |

All the Models were adjusted for age, sex, ethnicity, education level, Townsend deprivation index, smoking status, alcohol consumption, BMI, IPAQ activity group, eGFR, diabetes, dyslipidemia, CVD, and hypertension.

Supplementary Table 14. Sensitivity analyses of the associations between immune-inflammatory markers and all-cause mortality among patients with HF

| Analysis  (N = 1636) | HR (95% CI) | | | | |
| --- | --- | --- | --- | --- | --- |
|  | Quintile 1 | Quintile 2 | Quintile 3 | Quintile 4 | Quintile 5 |
| SII | 1.00 (ref.) | 0.86 (0.73, 1.01) | 0.95 (0.81, 1.12) | 1.06 (0.90, 1.26) | 1.13 (0.94, 1.35) |
| SIRI | 1.00 (ref.) | 1.02 (0.87, 1.20) | 1.17 (0.99, 1.37) | 1.38 (1.17, 1.63) | 1.45 (1.21, 1.74) |
| AISI | 1.00 (ref.) | 1.03 (0.88, 1.21) | 1.14 (0.97, 1.35) | 1.16 (0.98, 1.37) | 1.31 (1.10, 1.56) |
| IBI | 1.00 (ref.) | 0.99 (0.84, 1.16) | 1.07 (0.91, 1.26) | 1.32 (1.11, 1.56) | 1.27 (1.04, 1.56) |
| CALLY | 1.00 (ref.) | 0.95 (0.78, 1.15) | 0.80 (0.66, 0.97) | 0.73 (0.60, 0.88) | 0.78 (0.64, 0.95) |
| NLR | 1.00 (ref.) | 0.95 (0.81, 1.12) | 1.05 (0.89, 1.24) | 1.27 (1.08, 1.50) | 1.26 (1.06, 1.50) |

All the Models were adjusted for age, sex, ethnicity, education level, Townsend deprivation index, smoking status, alcohol consumption, BMI, IPAQ activity group, eGFR, diabetes, dyslipidemia, CVD, and hypertension.

Supplementary Table 15. Incremental predictive value of immune-inflammatory markers for incident HF assessed by IDI and NRI based on Model 3

| Marker | IDI (95% CI) | *P* value | NRI (95% CI) | *P* value |
| --- | --- | --- | --- | --- |
| SII | 0.0177 (0.0159, 0.0212) | <0.001 | 0.0419 (−0.0354, 0.0637) | 0.44 |
| SIRI | 0.0222 (0.0181, 0.0272) | <0.001 | −0.0344 (−0.0891, 0.0162) | 0.32 |
| AISI | 0.0159 (0.0121, 0.0195) | <0.001 | −0.0738 (−0.1175, 0.0212) | 0.28 |
| IBI | 0.0450 (0.0387, 0.0529) | <0.001 | 0.1596 (0.0871, 0.2247) | <0.001 |
| CALLY* | 0.0412 (0.0353, 0.0494) | <0.001 | −0.0039 (−0.0062, −0.0026) | <0.001 |
| NLR | 0.0257 (0.0221, 0.0296) | <0.001 | 0.0371 (−0.0133, 0.0896) | 0.35 |

*For the CALLY index, values were inverted (multiplied by −1) for comparison.

Abbreviations: IDI, integrated discrimination improvement; NRI, net reclassification improvement;

Model 3 included age, sex, ethnicity, education level, Townsend deprivation index, smoking status, alcohol consumption, BMI, IPAQ activity group, eGFR, diabetes, dyslipidemia, CVD, and hypertension.


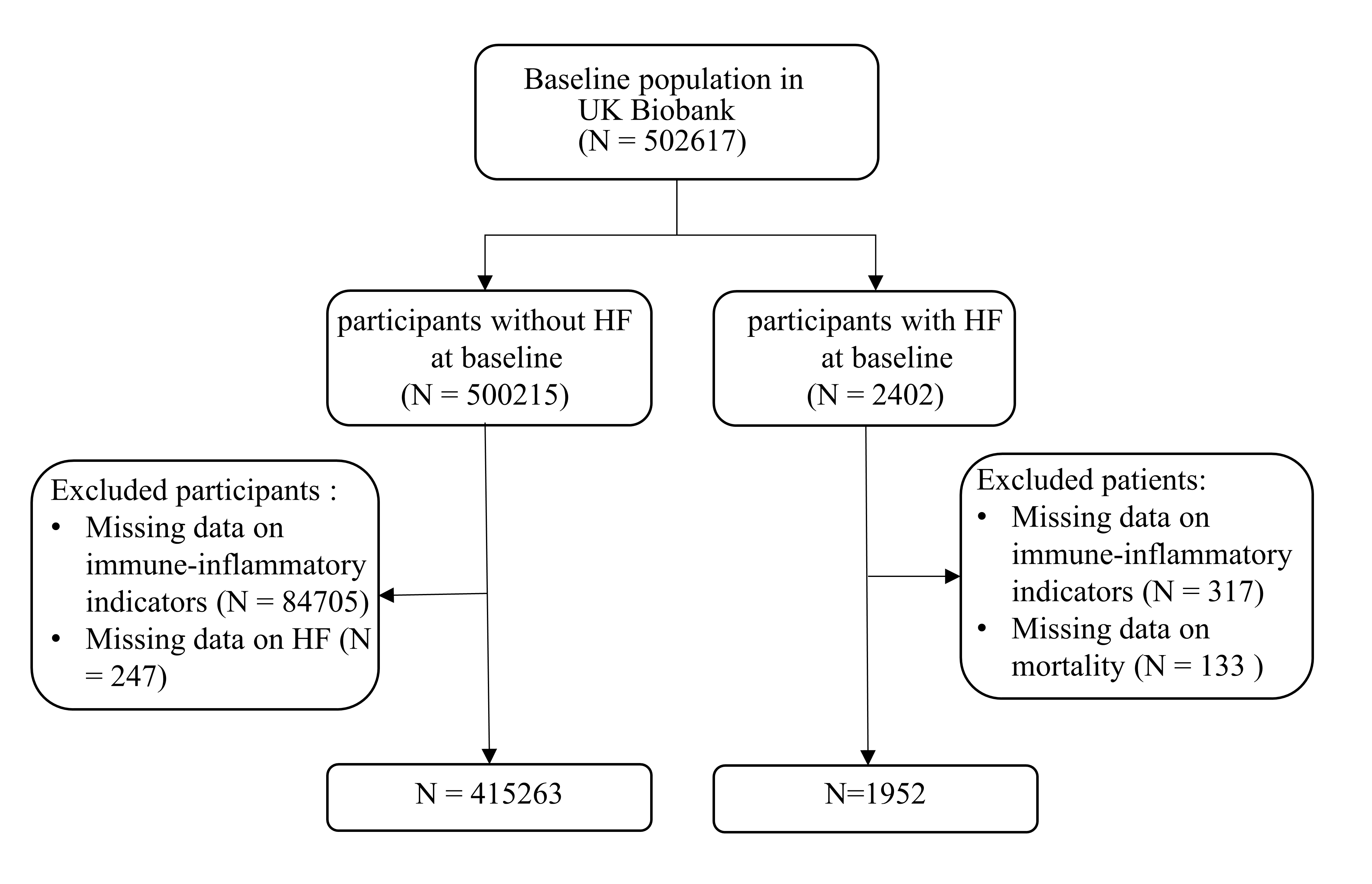


Supplementary Figure 1. Flow chart of participants included in the present UK Biobank study
